# Danish Medical Institutions

**Published:** 1903-11-21

**Authors:** 


*To be continued.*

### DANISH MEDICAL INSTITUTIONS.

(*Continued from page* 23.)

### II.—THE PRIVATE CLINICS.

An outstanding feature of hospital life in Denmark is the large number of private pay hospitals, or, as they are called, "cliniques," which exist. Many doctors have their own, and in the suburbs of Copenhagen it is quite common to see a plate which states that the house to whose door or gate it is affixed is Dr. A.'s or Dr. G.'s clinic. Of late years, however, nurses are beginning also to set up clinics, to which doctors who have not hospitals of their own send patients.

It may seem that there is nothing in this which differs greatly from our English custom. The difference really lies in the comparatively trivial cases which are treated away from home. Few people here go into a nursing home when they have rheumatism or bronchitis; our kindred institutions are, speaking generally, reserved for severe surgical cases. In Denmark there is no such limitation. Hospital treatment is the rule rather than the exception. This is due to the fact that the bulk of the people live in flats, and if the fashion of doing so continues to spread in England at anything like its present rate, there can hardly be a doubt that it will soon or late be followed by a corresponding increase in the number of pay hospitals. Even now the want of these is greatly felt by people of moderate means.

Denmark is a country of moderate means. The really rich are few, though it may be an exaggeration to say that they could all be counted on one's fingers. I was told this, but do not take it literally. Still, it is certain that people live there contentedly and comfortably on incomes that would seem despicable to an English family of a corresponding position. This is only partly due to greater cheapness of rent and food, while clothing is distinctly dearer. But the people are content with simpler things. Fewer servants are kept, the mistress of the house takes a more active part in the work of it and a more intelligent interest in its economy, and makes her gowns last for years instead of months. One could easily count the number of private carriages in Copenhagen, and a medical man in good practice is content to live in a modest flat and never dreams of keeping a carriage. Why should he? Has he not a bicycle?—and Denmark, with its level plains, is made for bicycling. Or can he not make use of the admirable electric tramcars which Copenhagen possesses, in which, by means of transfer tickets, one can go from one end of the city to another for a fraction over a penny? If he were told that it is desirable to keep a carriage in order to simulate a prosperity which he does not possess but which, by means of the deception, he hopes to attain, he would not laugh—for the Danes are, on the whole, a serious people, though their seriousness is the calm gravity of people who look upon life as a whole, not the depressed gloom of people who are ever striving against its conditions. But he would certainly wonder how it made for prosperity to spend more than one could afford. They do not "keep up appearances" in Denmark.

I have said that living in flats tends to make people more ready to go to a hospital when they are ill, and as each room in a Danish flat opens into those on either side, it is obvious that quiet and isolation are practically unobtainable in it. Add to this that Copenhagen is a very noisy city. It is paved throughout with granite "setts," and as people there go to bed late and get up early—for indeed in that stimulating air one does not want much sleep—the noise of traffic never seems to cease. The bells of the electric cars are clanging all day long and far into the night, and at the most peaceful hour of the twenty-four, there is always a cab or cart coming clattering over the stone-paved street. Only in the suburban clinics, hidden in side streets and nestling in gardens, can the silence which invalids need be found. And the clinics are practically within the means of all but the really poor, who go to the hospitals. A lady of my acquaintance was treated at the clinic of a well-known doctor for five kroner a day—about 5s. 6d. This charge included not only board and lodging, but nursing, hot-air treatment and doctor's fees! The only "extra" was an electric bath. For this price she had a private room; had she been willing to share a room with two other patients, the charge would have been only three kroner a day. It is probable, however, that when a doctor owns the clinic, so that he can practically see all his patients under one roof, the charges are less than when he has to go to see them at a clinic owned by some other person. I went to the newest clinic in Copenhagen, that of Froken Birgitta Lind, in Bakkersgade, which was opened only in March of this year. It is an admirably appointed hospital with a good operating room, and every convenience for treating severe surgical cases. There the charges ran from four to eight kroner a day.

Even for obstetric cases there is a pay hospital. When I expressed surprise at this I was asked if we had no lying-in hospitals in England, and it was rather difficult to explain—especially to the friends and relations of a baby which had just seen the light in the "Fydethemmer" at Fredericksberg, the kind of cases which so largely fill our obstetric hospitals here. As this home is subsidised by the commune of Fredericksberg, the inhabitants of that suburb pay only two kroner, fifty ore a day (rather more then half-a-crown) for a bed in a ward containing three beds, and six kroner for a private room, while patients from Copenhagen—which is within a penny tram ride—or any other part, pay five kroner a day in the ward and eight in a private room. In each case fifteen kroner is charged in addition for the actual confinement. It must be remembered that this institution is meant for normal cases, not specially for such as promise difficulties which would make an English doctor suggest the patient going into a hospital. As evidence of this I may mention that the stay of each patient is limited to a fortnight, and only when obvious danger would attend removal is a longer residence permitted. The number of patients treated every year averages 150, and the maternal deaths two.

